# Micro-scale vineyard variation influences phenolic composition and color expression in ‘cabernet sauvignon’ wines

**DOI:** 10.1016/j.fochx.2026.104751

**Published:** 2026-11-04

**Authors:** Xiao Meng, Hualin Zhang, Xuechen Yao, Yibin Lan, Tonghua Shi, Yanzhi Cui, Changqing Duan, Guangfeng Wu, Qiuhong Pan

**Affiliations:** a Center for Viticulture and Enology, College of Food Science and Nutritional Engineering, China Agricultural University, Beijing 100083, China; b Key Laboratory of Viticulture and Enology, Ministry of Agriculture and Rural Affairs, Beijing 100083, China; c Longus Vineyard Co., Ltd., Changli 066600, Hebei, China

**Keywords:** Pyranoanthocyanins, Terroir, Phenolic profiling, Soil nutrients, Pigment evolution, Precision viticulture

## Abstract

Anthocyanin-derived pigments contribute to red wine color, but how variation among neighboring vineyard plots is reflected in pigment evolution remains unclear. Using LC-MS and colorimetry, we profiled ‘Cabernet Sauvignon’ grapes and wines from four neighboring plots within one vineyard in China's Jieshi Mountain region across 2020 and 2021. In 2020, higher a* and C*_ab_ in CS1 and CS3 were associated with polymeric pigments and pyranoanthocyanins, respectively. In the wetter 2021 vintage, wines contained more monomeric anthocyanins but negligible polymeric pigments and showed a yellower color (higher b* and *hab*). PLS regression associated a* and C*_ab_ with anthocyanin-derived pigments, whereas b* and *hab* were related to flavonols. Epicatechin, malvidin-3-*O*-acetylglucoside, and malvidin-3-*O*-coumaroylglucoside recurred as candidate plot indicators and showed exploratory soil associations. Overall, plot-related variation remained detectable despite vintage modulation, while berry-to-wine changes indicated that color reflected anthocyanin transformation during fermentation and stable pigment formation rather than monomeric anthocyanin abundance alone.

## Highlights

• Vineyard soil differences shape red wine color and visual appearance.
• Wine color depends more on pigment composition than total anthocyanin amount.
• Vineyard-specific phenolic patterns remain detectable from grapes through fermentation to wine.
• Soil organic matter and nutrients influence key grape color-related compounds.
• Stable phenolic compounds can indicate vineyard origin across different years.

## 1 Introduction

Color is one of the most important sensory attributes of red wine, profoundly influencing consumer perception, product typicity, and market value (Galaz-Torres et al., 2025). Beyond their coloring functions, some natural pigments possess potential biological activities, while toxicological concerns associated with certain synthetic food dyes have further increased interest in natural colorants (Damotharan et al., 2024; Mattioli et al., 2020; Priyanka & Karthick Raja Namasivayam, 2026). In young red wines, color is primarily determined by anthocyanins and their derivatives, which originate from grape berry skins and undergo complex transformations during fermentation. These transformations include the formation of pyranoanthocyanins and polymeric pigments, contributing to long-term color stability and visual appeal (Yao et al., 2024; Zhang et al., 2021).

Anthocyanin accumulation in grape berries is highly sensitive to environmental and viticultural factors such as temperature, light exposure, and water availability during ripening (Lu et al., 2025; Tian et al., 2025; Yao, Zhang, et al., 2024). Among these factors, soil texture, organic matter, water-holding capacity, and cation exchange capacity are particularly influential, as they regulate vine vigor, berry size, and phenolic biosynthesis (Palma-López et al., 2024; Shi et al., 2025). While anthocyanins are the dominant pigments in young red wines, color development is also modulated by co-pigmentation with flavonols and flavan-3-ols, as well as by the formation of derived pigments such as pyranoanthocyanins (Li & Duan, 2019; Zhang et al., 2022). The stability and hue of wine color thus depend not only on the quantity but also on the structure and reactivity of phenolic compounds.

Although terroir studies have linked soil, slope, and climate to grape and wine phenolic profiles, plot-scale variation in anthocyanin-derived pigments remains insufficiently characterized, particularly in China (Del-Castillo-Alonso et al., 2016; Lu et al., 2025). Previous Chinese terroir studies have primarily compared major wine-producing regions or officially defined subregions (Lan et al., 2022; Zhao et al., 2023), whereas phenolic and color variation among neighboring plots within a single commercial vineyard remains less well characterized. Although plot-scale differences in aroma profiles have been reported for the same Jieshi Mountain vineyard (Chen et al., 2023), corresponding variation in phenolic composition, particularly anthocyanin-derived pigments, remains unclear.

In this study, we compared ‘Cabernet Sauvignon’ grapes and wines from four neighboring plots within a large commercial vineyard across the 2020 and 2021 vintages. By integrating phenolic profiling, CIELAB color parameters, and soil and climate data, we assessed (i) plot- and vintage-related variation in grape and wine phenolic composition and color, (ii) associations between pigment classes and color attributes, (iii) continuity of candidate phenolic signatures from berries to wines, and (iv) exploratory relationships between soil properties and berry phenolics. This design provides a plot-scale assessment of how vineyard heterogeneity is reflected in phenolic and color profiles.

## 2 Materials and methods

### 2.1 Experimental plots, climate, and soil conditions

The study was conducted in four *Vitis Vinifera*, L. Cabernet Sauvignon vineyard plots located in Changli County, Hebei Province, China (119.78°E, 39.93°N). The four adjacent commercial vineyard plots differed in area, relative position, and planting year. CS1 was located in the northwestern part of the vineyard and covered 8.54 ha (planted in 2001); CS2 was located in the northeastern and eastern part and covered 5.69 ha (planted in 2004); CS3 was centrally located and covered 4.94 ha (planted in 2006); and CS4 was located in the eastern part and covered 7.14 ha (planted in 2011). All plots were situated along the same gentle slope (approximately 0.3%), with CS1 at the upper position and CS2 at the lower position. The vines were trained using a vertical shoot-positioning system, with a spacing of 2 m × 1 m and north–south-oriented rows. No irrigation was applied during the growing season. Detailed information regarding vineyard characteristics, experimental design, sampling procedures and management practices has been reported previously (Chen et al., 2023).

The study covered two consecutive vintages (2020 and 2021). Although effective accumulated temperature during both the growing season (April–September) and the month preceding harvest was comparable between vintages, precipitation was substantially higher in 2021, particularly during September when rainfall was approximately 2.7-fold greater than that recorded in 2020. Despite their close geographical proximity, the four vineyard plots exhibited distinct soil characteristics. Within each plot, nine spatially distributed sampling locations were divided into three groups, each comprising three locations. At each soil depth, soil collected from the three locations within each group was thoroughly composited to generate one independent composite sample. Thus, three composite soil replicates were obtained per plot at each of the three depth intervals: topsoil (T, 0–30 cm), intermediate soil (I, 30–60 cm), and deep soil (D, 60–90 cm). Detailed climatic and soil data have been reported previously (Chen et al., 2023).

### 2.2 Grape berry sampling and physicochemical analysis

Grape berries from all four vineyard plots were harvested on the same date within each vintage, on 6 October 2020 and 2 October 2021, respectively. Each vineyard plot was divided into three spatially separated sampling zones, which served as three independent within-plot biological replicates. Within each zone, berries were collected using a five-point sampling scheme. Clusters were randomly selected from both the sun-exposed and shaded sides of the rows, and six berries were collected from spatially distributed positions on each selected cluster (upper, lower, left, right, front, and back) until 200 berries were obtained. Of these, 100 berries were randomly selected for physicochemical analysis, while the remaining 100 berries were immediately frozen in liquid nitrogen and stored at −80 °C for metabolite analyses, including phenolic profiling. The three biological replicates were processed and analyzed separately, resulting in a total of 600 berries collected from each plot.

For each biological replicate, 100 berries were weighed collectively to determine the hundred-berry weight and then manually pressed to obtain juice for physicochemical analysis. Total soluble solids (TSS) were measured using a digital refractometer (PAL-1, ATAGO, Tokyo, Japan) and expressed as °Brix. Juice pH was determined using a calibrated pH meter (FE20, Mettler Toledo, Greifensee, Switzerland). Titratable acidity (TA) was determined by titration with 0.1 mol/L NaOH to the phenolphthalein endpoint and expressed as g/L tartaric acid.

### 2.3 Wine fermentation and sample collection

For each vineyard plot, two independent 240 kg grape lots were collected across the plot and vinified separately in two 300 L stainless-steel fermenters (Chen et al., 2023). A 6% sulfurous acid solution was added to obtain a final SO₂ addition of 55 mg/L. Alcoholic fermentation (AF) was initiated by adding 200 mg/L yeast (Zymaflore FX10) and 40 mg/L pectinase (LAFASE LE GRAND CRU). The fermentation temperature was maintained at 20–25 °C. The cap was punched down every 8 h, and the temperature and specific gravity were monitored throughout AF. Alcoholic fermentation was considered complete when the specific gravity had decreased to approximately 0.990 and remained stable.

After alcoholic fermentation, the wine was separated from the pomace. Wines originating from the two fermenters were kept separate and transferred to 50 L glass vessels for MLF. Each vinification lot was inoculated with lactic acid bacteria (LACTOENOS B7 DIRECT 25HL), and the MLF temperature was maintained at 18–20 °C. The yeast, pectinase, and lactic acid bacteria were obtained from LAFFORT (Bordeaux, France).

MLF was monitored daily by paper chromatography from the fifth day after inoculation, with disappearance of the malic acid spot used as the preliminary endpoint criterion. At the end of MLF, the completion of MLF was further evaluated by measuring malic and lactic acid concentrations using a Fourier-transform infrared wine analyzer.

At the end of AF and MLF, one 750 mL sample was collected from each of the two separately vinified lots, and a third pooled sample was prepared by mixing equal volumes of wine from the two lots. Thus, the three measured wine samples from each plot comprised two samples from independently vinified fermenters and one pooled composite sample. The pooled sample represented the plot-level blend and was not considered a third independent vinification replicate.

Wine samples were centrifuged at 4000 ×*g* for 8 min before analysis. The pH, volatile acidity, titratable acidity, residual sugar, ethanol, malic acid, and lactic acid were determined using a Fourier-transform infrared wine analyzer (WineScan, FOSS Electric A/S, Hillerød, Denmark).

### 2.4 Extraction of phenolics from grape skins

Anthocyanins and flavan-3-ols were extracted from grape skins according to the methods of Wang et al. (2021) and Yao, Zhang, et al. (2024), with minor modifications.

For anthocyanin extraction, 0.1 g of freeze-dried grape skin powder was transferred to a 2.5 mL centrifuge tube, followed by the addition of 1 mL of 50% (*v*/v) methanol. The mixture was sonicated at 20 °C for 20 min, protected from light, and then centrifuged at 8000 rpm for 10 min at 4 °C. The supernatant was collected into a new 2.5 mL centrifuge tube. The residue was re-extracted with 1 mL of 50% methanol following the same procedure. The two extracts were combined, thoroughly mixed, and stored at −80 °C until analysis.

For flavan-3-ol extraction, 0.1 g of freeze-dried grape skin powder was weighed into a 5 mL centrifuge tube, and 1 mL of 70% acetone (containing 0.5% ascorbic acid) was added. The mixture was vortexed thoroughly and centrifuged at 10,000 rpm for 15 min at 4 °C. The supernatant was transferred to a new 5 mL centrifuge tube. This extraction was repeated three times, and the combined supernatants were collected. An aliquot of 400 μL was transferred to a 1.5 mL centrifuge tube and dried under a nitrogen stream at room temperature. The residue was reconstituted in 200 μL of methanol containing 1% HCl and neutralized with 200 μL of 200 mmol/L sodium acetate solution. The final solution was stored at −80 °C until analysis.

### 2.5 Analysis of phenolics in grape

Phenolic compounds in grape berries were analyzed by HPLC-MS/MS according to the method of Yao, Wu, et al. (2024), with minor modifications. An Agilent 1200 series HPLC system coupled to a 6410 triple quadrupole (QqQ) mass spectrometer was used, equipped with a Poroshell 120 EC-C18 column (150 mm × 3.0 mm, 2.7 μm). The column temperature was maintained at 55 °C. The mobile phase consisted of (A) 0.1% formic acid in water and (B) 0.1% formic acid in methanol–acetonitrile (50:50, *v*/v). The flow rate was 0.4 mL/min, and the injection volume was 5 μL. Prior to analysis, the extracts were filtered through a 0.22 μm organic membrane filter.

For anthocyanins, the gradient program was as follows: 90%–0% A (10%–100% B) over 15 min, followed by a 5-min post-run. For non-anthocyanin phenols, the gradient program was: 0–28 min, 90%–54% A (10%–46% B); 28–29 min, 54%–90% A (46%–10% B); followed by a 5-min re-equilibration at 90% A. Mass spectrometric detection was performed using an electrospray ionization (ESI) source in multiple reaction monitoring (MRM) mode. Anthocyanins were analyzed in positive ion mode, and non-anthocyanin phenols in negative ion mode. The source parameters were: spray voltage, 4 kV; ion source temperature, 150 °C; drying gas temperature, 350 °C; drying gas flow, 12 L/min; nebulizer pressure, 35 psi.

Compounds with available authentic standards were confirmed by matching their retention times and characteristic MRM transitions. Compounds lacking authentic standards, particularly anthocyanin-derived pigments, were putatively identified based on their chromatographic behavior, characteristic precursor/product-ion transitions, the in-house HPLC-MS/MS fingerprint database, and previously published data (Zhang et al., 2020). Quantification was performed using external calibration curves constructed in the present study. Detailed information on the MRM transitions, retention times, calibration ranges, regression equations, R^2^ values, quantification standards, and identification basis is provided in Table S2. The results were expressed as mg/kg berry fresh weight, converted from the dry skin concentration using the ratio of dry skin weight to berry fresh weight.

### 2.6 Analysis of phenolics in wine

Phenolic compounds in wine were analyzed following the method of Zhang et al. (2020), using the same HPLC-MS/MS instrument, column, mobile phase, and MS source parameters described above. Wine samples were filtered through a 0.22 μm aqueous membrane filter prior to injection. The chromatographic and mass spectrometric conditions for anthocyanins and non-anthocyanin phenols were identical to those used for grape berries.

For anthocyanin-derived pigments, the gradient program was: 0–1 min, 100% A; 3 min, 25% B; 15 min, 30% B; 20–25 min, 100% B; followed by a 5-min post-run. The flow rate was 0.3 mL/min, the column temperature was 55 °C, and the injection volume was 5 μL. The mass spectrometer was operated in positive ion mode.

Phenolic compounds were quantified using external calibration curves constructed with the corresponding authentic standards when available or with structurally related analogues when authentic standards were unavailable. In particular, anthocyanin-derived pigments were quantified using malvidin-3*-O-*glucoside as the representative standard and expressed as malvidin-3*-O-*glucoside equivalents. The standard assigned to each analyte, together with the compound-specific identification and quantification information, is provided in Table S2.

### 2.7 Measurement of color index

Color indices were measured as described previously (Zhang et al., 2024). The luminance value L*, the red-green value a*, and the yellow-blue value b* were determined using a CM-3700d spectrophotometer (Konica Minolta, Shanghai, China). Prior to analysis, wine samples were filtered through a 0.45 μm polyethersulfone (PES) membrane and transferred to a 2-mm cuvette. All samples were analyzed in triplicate. Additionally, the Chroma (C*_ab_) and Hue Angle (*h*_*ab*_) were calculated according to the following equation.

(1) $$C_{\mathrm{ab}}^{*}={\left({\left(a^{*}\right)}^{2}+{\left(b^{*}\right)}^{2}\right)}^{1/2}$$

(2) $$h_{\mathrm{ab}}=\tan^{-1}\left(b^{*}/a^{*}\right)$$

### 2.8 Statistics and data analysis

Liquid chromatography-mass spectrometry (LC-MS) data were processed using Agilent MassHunter software, and the concentrations of individual compounds were calculated based on calibration curves and expressed as mass concentrations. Data collation and preliminary processing were conducted in Microsoft Excel 2019. Partial least squares discriminant analysis (PLS-DA) was conducted using SIMCA 14 (Umetrics, Umeå, Sweden).

Color parameters (L*, a*, b*) measured during fermentation were imported into Adobe Photoshop CS2018 (Adobe Systems Inc., San Jose, CA, USA), and approximate color palettes were reconstructed to simulate visual wine color evolution.

Statistical analyses were performed using R software (version 4.1.3), and analysis of variance was conducted using the ‘agricolae’ package. One-way ANOVA was used to compare vineyard plots within each vintage and sampling or fermentation stage. Two-way ANOVA was used to evaluate the effects of vintage, vineyard plot, and their interaction on the concentration of each phenolic compound. Vintage and vineyard plot were treated as fixed factors, and berry, AF-wine, and MLF-wine datasets were analyzed separately. Statistical significance was defined as *P* < 0.05.

PLS-DA of berry and wine phenolic profiles and PLSR relating wine phenolic composition to color parameters were performed using SIMCA 14.0 (Umetrics, Umeå, Sweden). Wine-based models included three measured samples per plot, comprising two fermenter-specific samples and one equal-volume pooled composite, resulting in 12 observations per vintage. The pooled sample was assigned to its corresponding plot but was not regarded as a third independent vinification replicate. Variables were mean-centered and unit-variance scaled. Model performance was assessed using R^2^X(cum), R^2^Y(cum), and Q^2^(cum), with Q^2^ calculated by seven-fold cross-validation; the retained components are reported in the corresponding figure captions. Variables with VIP values >1 were considered relevant to plot discrimination. PLSR models were further assessed using 500 permutation tests.

Heatmaps were generated using TBtools v2.422, compositional bar plots were prepared using GraphPad Prism v9.4.1, and network graphs were constructed using Cytoscape v3.8.0. Statistical significance was assessed with a threshold of *p* < 0.05.

## 3 Results

### 3.1 Vineyard plot-dependent variation in berry and wine physicochemical and color characteristics

The physicochemical characteristics of grape berries and wines are summarized in Fig. S1. Some of these data were obtained from the same experimental batches reported previously (Chen et al., 2023) and are included here only to provide compositional context for the subsequent phenolic and color analyses. Total soluble solids (TSS) did not differ significantly among the four plots within either vintage (Fig. S1), indicating broadly comparable technological maturity at harvest, although berry weight, pH, and titratable acidity exhibited some plot-associated variation. Grapes from CS1 and CS4 generally exhibited higher pH values than those from CS2 and CS3 (Fig. S1). Differences among plots were also observed in berry weight, must composition, and wine physicochemical parameters following AF and MLF (Fig. S1). In the final MLF wines, malic acid was below the reporting limit of the WineScan method (Table S1), supporting comparable completion of MLF across vineyard plots and vintages. Taken together, the four vineyard plots produced grapes and wines with broadly comparable maturity and basic compositional attributes, providing a suitable framework for evaluating plot-dependent differences in phenolic composition and color expression.

To further characterize differences among vineyard plots and vintages, wine color parameters were evaluated using the CIELAB color system. Wine color parameters showed markedly greater variation among vineyard plots and vintages than between fermentation stages (AF and MLF) (Fig. 1).

**Fig. 1:**
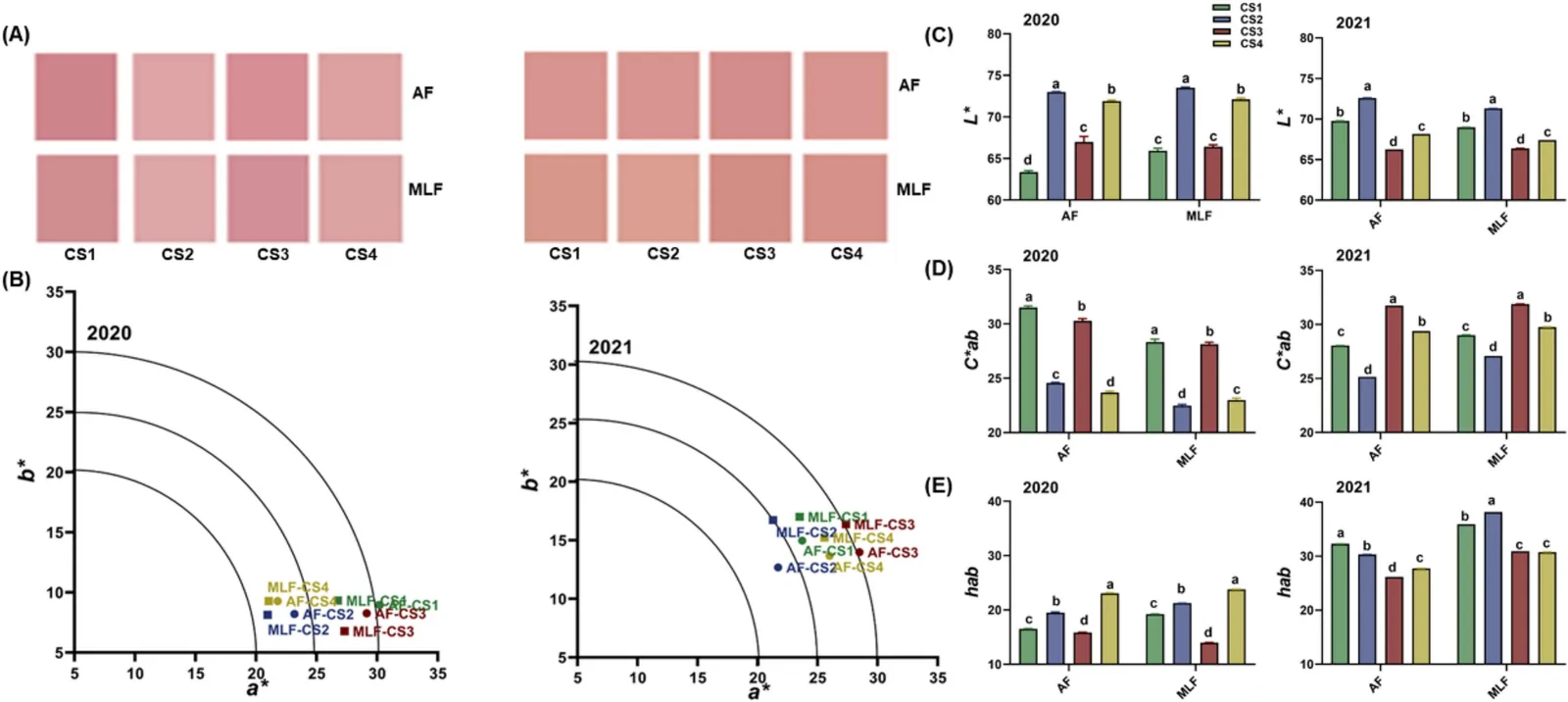
Color characteristics of Cabernet Sauvignon wines from four vineyard plots after alcoholic fermentation (AF) and malolactic fermentation (MLF) in the 2020 and 2021 vintages. (A) Reconstructed wine color palettes based on measured CIELAB color parameters (L*, a*, and b*). (B) Distribution of AF and MLF wine samples in the a*-b* color space. (C) L* (lightness), (D) C*_ab_ (chroma), and (E) *hab* (hue angle) values. Data are presented as mean ± standard deviation (*n* = 3). Different letters indicate significant differences among vineyard plots within the same vintage and fermentation stage (*P* < 0.05).

Clear differences were observed between the two vintages. Wines produced in 2020 generally exhibited deeper red hues, whereas those from 2021 displayed a more yellow-toned appearance, as illustrated by the reconstructed color palettes and the distribution of samples in the a*-b* color space (Fig. 1a and b). Across all plots, 2021 wines tended to exhibit higher b* and *h*_*ab*_ values than their 2020 counterparts (Fig. 1b and e), indicating a shift toward yellower color tones.

Within each vintage, distinct plot-dependent color patterns were also evident (Fig. 1b-e). In 2020, wines from CS1 and CS3 were characterized by higher a* and C*_ab_ values, indicating greater red color intensity and saturation, whereas CS2 and CS4 exhibited higher L* and *h*_*ab*_ values, corresponding to lighter and less saturated color expression. In contrast, the color pattern shifted in 2021, with CS3 and CS4 showing higher a* and C*_ab_ values, while CS1 and CS2 were associated with elevated b*and *h*_*ab*_ values and a more pronounced yellow hue.

These results indicate that both vineyard plot and vintage substantially influenced wine color expression, whereas the transition from AF to MLF resulted in comparatively minor changes. The pronounced color divergence among plots and vintages suggests underlying differences in phenolic composition and pigment evolution, which were further investigated in subsequent analyses.

### 3.2 Relationships between phenolic composition and wine color attributes

To identify the phenolic compounds contributing to color variation among plots and vintages, partial least squares regression (PLSR) was performed using wine color parameters (L*, a*, b*, C*_ab_ and *h*_*ab*_) as response variables and phenolic compounds measured after AF and MLF as explanatory variables (Fig. 2). In 2020, the PLSR model achieved R^2^X(cum) = 0.869, R^2^Y(cum) = 0.979, and Q^2^(cum) = 0.959; in 2021, R^2^X(cum) = 0.368, R^2^Y(cum) = 0.822, and Q^2^(cum) = 0.742. The 500-permutation tests yielded R^2^ and Q^2^ regression-line intercepts of 0.1587 and − 0.6222 for the 2020 model and 0.2256 and − 0.3240 for the 2021 model, respectively, indicating no evident overfitting (Fig. S2). Nevertheless, given the limited sample size, these models were interpreted primarily as exploratory descriptions of the associations between phenolic composition and wine color.

**Fig. 2:**
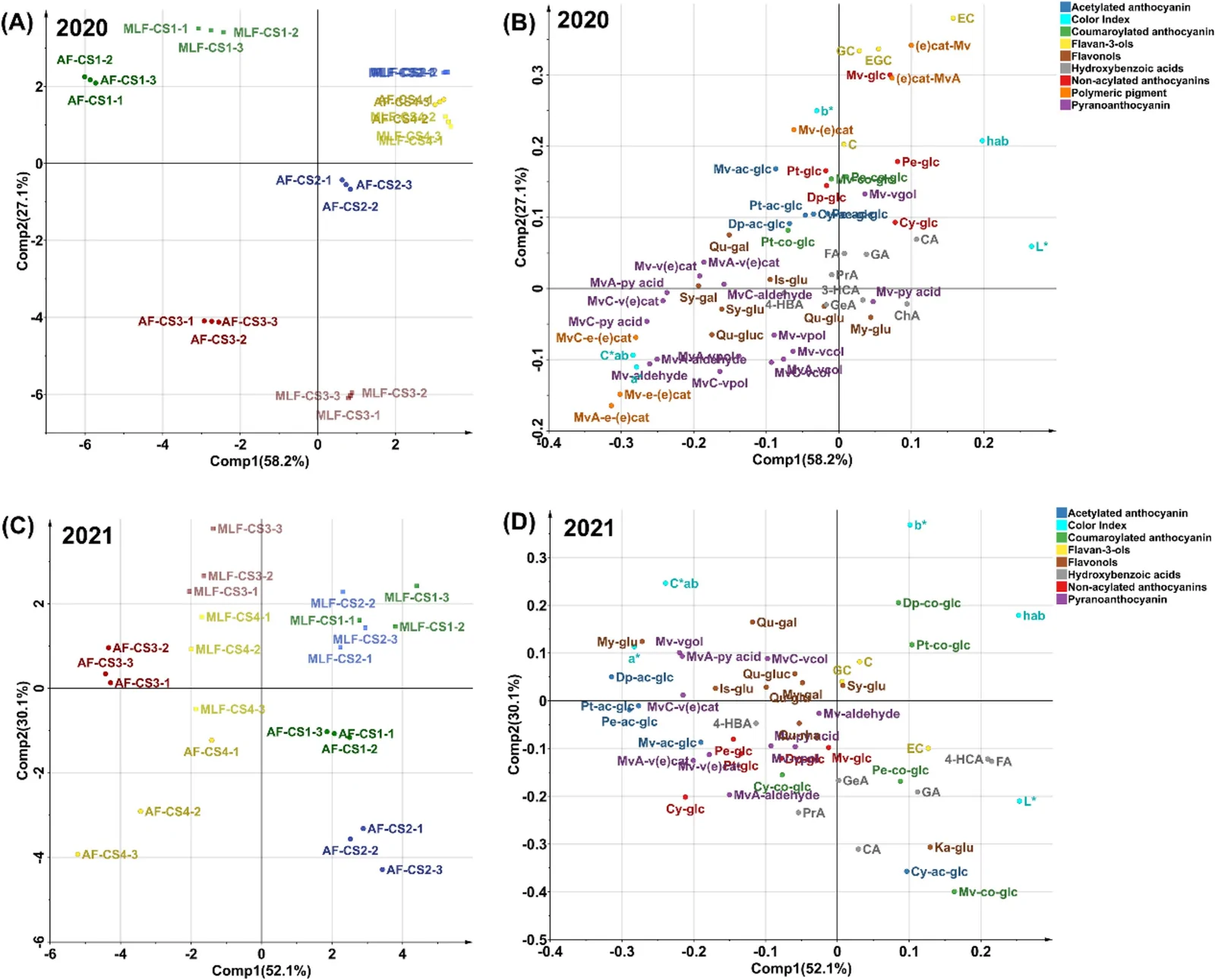
Color attributes and multivariate relationships between wine color and phenolic composition across four vineyard plots over two vintages. PLSR score plots based on wine color parameters and phenolic composition in 2020 (A) and 2021 (C), showing sample distributions by vineyard plot. PLSR loading plots showing the associations of individual phenolic compounds with wine color variation in 2020 (B) and 2021 (D). Color parameters include L* (lightness), a* (red–green), b* (yellow–blue), C*_ab_ (chroma), and *h*_*ab*_ (hue angle). The 2020 and 2021 PLSR models retained four and two components, respectively; the first two components are displayed in the score and loading plots. Each model included 12 wine observations per vintage, including one pooled composite sample from each plot. (For interpretation of the references to color in this figure legend, the reader is referred to the web version of this article.)

Across both vintages, a* and C*_ab_ were generally associated with anthocyanin-derived pigments, whereas b* and *h*_*ab*_ showed closer relationships with flavonols. In contrast, L* was negatively associated with several anthocyanin-derived pigments, indicating that the accumulation of these compounds contributed to darker and more saturated wine color.

The phenolic basis of color expression differed between vintages. In 2020, wines from CS1 and CS3, which exhibited higher a* and C*_ab_ values, were closely associated with total anthocyanins, vitisin A, vitisin B and A-e-F pigments. These compounds clustered near the positive direction of the a* and C*_ab_ vectors, suggesting an important contribution to red color intensity and saturation. Conversely, wines from CS2 and CS4 were associated with higher L* and *h*_*ab*_ values, corresponding to lighter and less saturated color expression. In 2021, a different pattern emerged, with the overall color tending toward yellow tones and polymeric pigments being barely detectable. Wines from CS3 and CS4 displayed higher a* and C*_ab_ values, characterized primarily by elevated levels of monomeric anthocyanins, as well as relatively high contents of pyranoanthocyanins and flavonols. In contrast, wines from CS1 and CS2 were positioned closer to the b* and *h*_*ab*_ vectors, featuring relatively high contents of flavanols and phenolic acids but limited abundance of anthocyanin-derived pigments. Overall, CS3 consistently exhibited higher a* and C*_ab_ values across both vintages, indicating that wine color expression was associated with vineyard plot.

These findings highlight that wine visual appearance depends not merely on anthocyanin concentration per se but on pigment composition, polymerization state, and pigmentation equilibrium between monomeric and polymeric pigments—all of which varied with both vintage and vineyard plot.

### 3.3 Identification of plot-discriminating phenolic signatures in berries and wines

In this study, 15 monomeric anthocyanins, 8 flavonols, and 3 flavan-3-ols were consistently detected in the skins of mature ‘Cabernet Sauvignon’ grapes over two vintages (Table S3). Two-way ANOVA revealed that most compounds were significantly affected by vintage, vineyard plot, and their interaction across the two growing seasons (Table S3). Notably, only a few phenolics showed limited sensitivity to either factor, for instance, Mv-glc was barely influenced by vineyard plot, while Qu-gal remained relatively stable across both vintage and location. In contrast, all five *p*-coumaroylated anthocyanins were highly responsive to both vintage and plot differences, highlighting the strong influence of environmental conditions on phenolic composition and its spatial variability.

Given the sensitivity of grape phenolic composition to environmental conditions, partial least squares discriminant analysis (PLS-DA) was performed to assess differences among the four vineyard plots (Fig. 3). In 2020, the score plot showed clear separation of CS1-CS4 samples along Comp1 (67.4%) and Comp2 (15.0%). In 2021, separation was evident along Comp1 (48.8%) and Comp2 (20.5%) (Fig. 3A-D). In both vintages, samples from CS2 were positioned on the left side of the score plots and were generally positively associated with a wide range of phenolic compounds. In contrast, CS4 consistently appeared on the right side of the x-axis and showed negative associations with most phenolics. Notably, CS1 was also located on the positive side in 2020 but differed from CS4 by exhibiting the highest total phenolic content, primarily due to its strong association with Mv-glc and its acylated derivatives. These compounds, being the most abundant anthocyanin components across all samples, were spatially distinct from many other phenolics, suggesting that their distribution pattern did not always mirror that of the broader phenolic matrix.

**Fig. 3:**
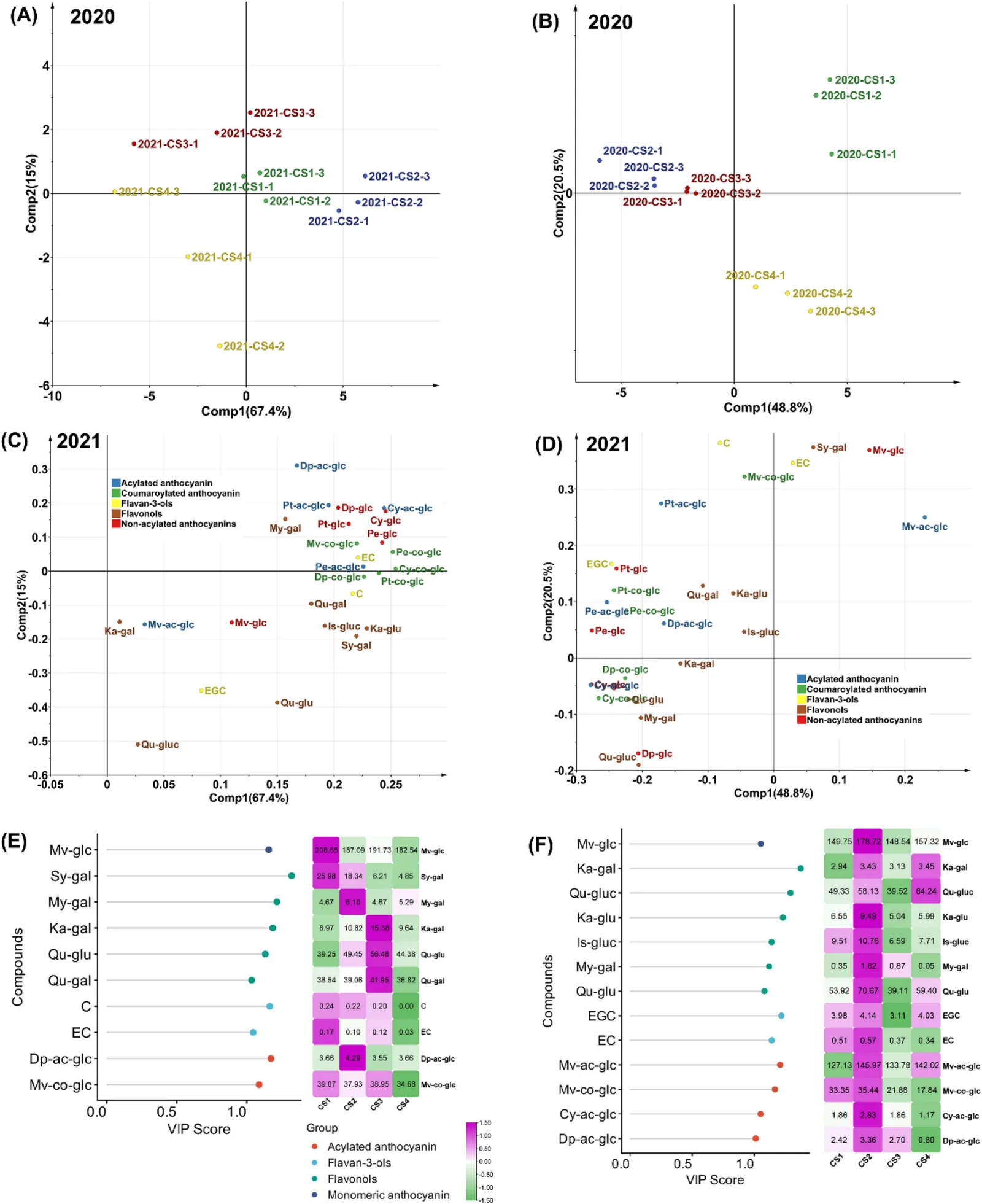
PLS-DA multivariate analysis of grape skin phenolic profiles from four vineyard plots in 2020 and 2021 vintages. PLS-DA multivariate analysis of grape skin phenolic profiles from four vineyard plots in the 2020 and 2021 vintages. (A, B) Score plots showing sample separation by vineyard plot in 2020 (A) and 2021 (B). (C, D) Corresponding loading plots indicating the phenolic compounds contributing to group discrimination in 2020 (C) and 2021 (D). (E, F) Key discriminant compounds with variable importance in projection (VIP) values >1 in 2020 (E) and 2021 (F). The left panels show the VIP scores, and the right panels show heatmaps of their concentrations (mg/kg berry fresh weight) across the four vineyard plots. The grape phenolic PLS-DA models for 2020 and 2021 retained six and seven components, respectively; the first two components are displayed in the score and loading plots.

Further analysis of variables with VIP > 1 revealed that flavonols were the most important contributors to the separation among vineyard plots (Fig. 3E and F). In 2020, five of the ten compounds with VIP > 1 were flavonols, while in 2021, six of thirteen fell into this class. My-gal, Ka-gal, and Qu-gluc were consistently among the key discriminating compounds across both vintages. In addition, acylated anthocyanins such as Mv-co-glc and Dp-ac-glc, the flavan-3-ols such as epicatechin (EC), and Mv-glc itself also contributed significantly to the observed plot differences. Notably, in 2021, CS2 exhibited the highest levels for nearly all VIP > 1 compounds, underscoring its distinct phenolic profile relative to the other plots. These results suggest that spatial variation in grape phenolic composition involves not only quantitative differences but also shifts in phenolic profiles across multiple compound classes.

To investigate how the phenolic differences observed in grape skins translated into wine composition, wines from two vintages (2020 and 2021) were analyzed following both AF and MLF. Two-way ANOVA was used to evaluate the effects of vintage, vineyard plot, and their interaction on the concentrations of individual phenolic compounds in AF and MLF wines (Table S4, Table S5). The 2020 wines exhibited greater phenolic diversity, characterized by abundant and varied polymeric pigments, whereas the 2021 wines contained negligible polymeric pigments but significantly higher concentrations of catechin (C) and gallocatechin (GC). Vineyard plot emerged as a statistically significant factor (*p* < 0.05) influencing nearly all phenolic classes, indicating that vineyard plot was significantly associated with wine phenolic composition.

Partial least squares discriminant analysis (PLS-DA) of wine phenolic profiles identified key discriminant compounds (VIP > 1) that distinguished the four vineyard plots (Fig. 4A–F). The loading plots (Fig. 4C and D) display only compounds with VIP > 1, whereas Fig. 4E and F present the compounds with VIP > 1. In 2020, the model explained 58.0% and 21.8% of the variance along Comp1 and Comp2, respectively; in 2021, the corresponding values were 36.5% and 27.9% (Fig. 4C and D). Notably, pyranoanthocyanins (Mv-vpol, Mv-vgol, MvA-vpol, and MvC-vpol)—stable pigments that confer red-orange hues to wine—were consistently associated with plot discrimination across both vintages (Fig. 4E).

**Fig. 4:**
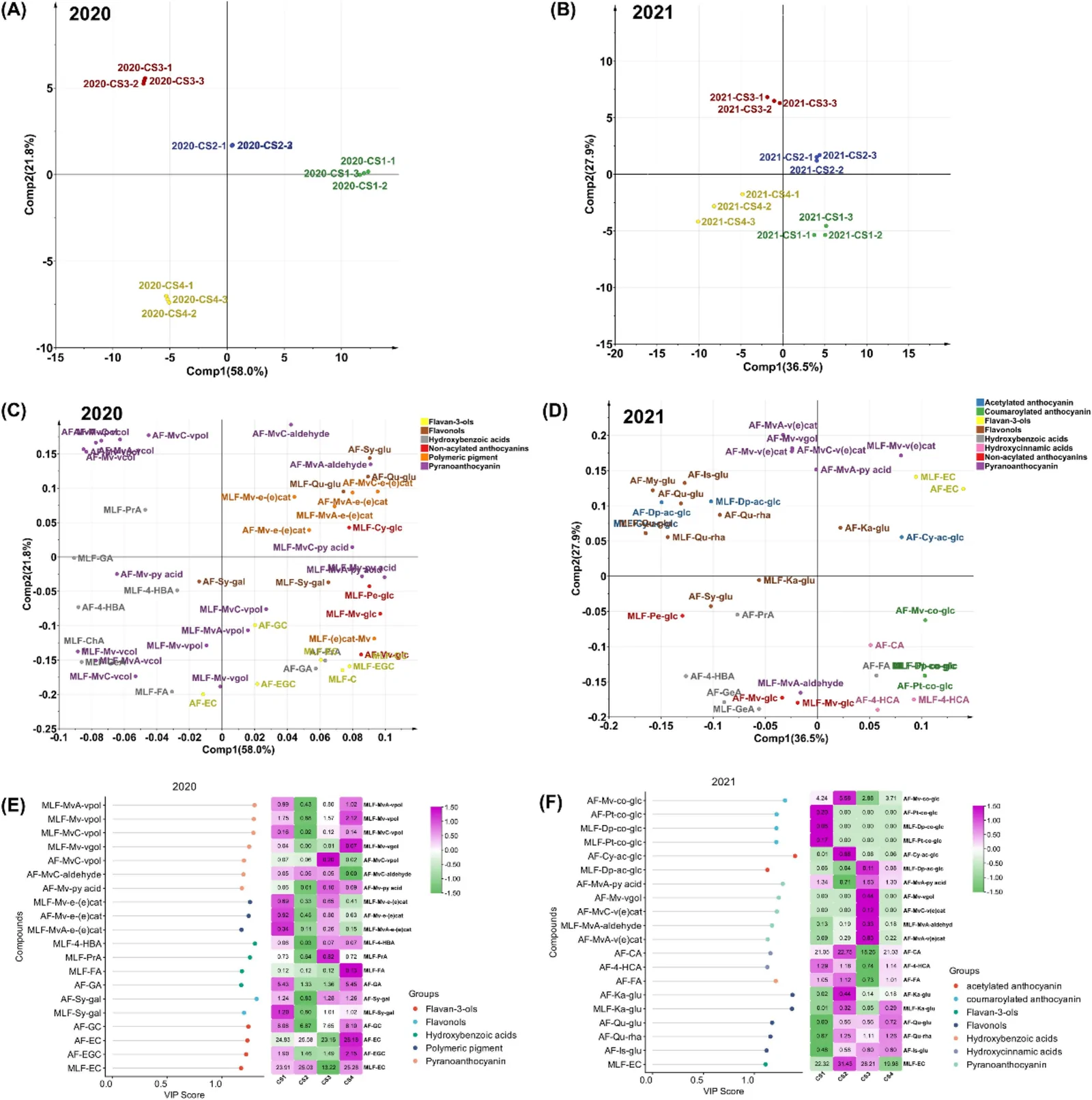
PLS-DA multivariate analysis of wine phenolic profiles from four vineyard plots in 2020 and 2021 vintages. (A, B) Score plots showing the separation of samples by vineyard plot in 2020 (A) and 2021 (B). (C, D) Corresponding loading plots indicating the phenolic compounds contributing to group discrimination in 2020 (C) and 2021 (D). (E, F) Key discriminant compounds with variable importance in projection (VIP) values >1 in 2020 (E) and 2021 (F). The left panel shows the VIP scores, and the right panel shows a heatmap of their contents (mg/L) across the four vineyard plots, enabling comparison of these critical compounds among the plots. The wine phenolic PLS-DA models for 2020 and 2021 retained three and four components, respectively; the first two components are displayed in the score and loading plots. Each model included 12 wine observations per vintage, including one pooled composite sample from each plot.

Spatial analysis of the 2020 loading plot revealed distinct clustering patterns among the plots (Fig. 4C). CS1 was associated with polymeric pigments in Quadrant 1, CS3 correlated closely with pyranoanthocyanins in Quadrant 2, and CS4 was linked to phenolic acids in Quadrant 3. These associations aligned with the color analysis results: CS1 and CS3 exhibited the most pronounced red coloration, as reflected by their higher a* values (Fig. 2B), indicating that polymerized pigments and pyranoanthocyanins were the pigment classes most strongly associated with wine redness in 2020. Polymeric pigments were abundant in 2020 but fell largely below the detection limit in 2021 and were therefore absent from the 2021 analysis. In 2021, CS3 remained closely associated with pyranoanthocyanins along the positive Y-axis (Fig. 4D) and displayed the highest C*_ab_ values (Fig. 1D), reinforcing the role of these stable pigments in maintaining color intensity under wetter vintage conditions. This discrepancy may be attributed to the clustering of CS2 in Quadrant 1 alongside high levels of flavan-3-ols (Fig. 4D), which contribute more to astringency than to chromatic properties. Meanwhile, CS1 was positioned in Quadrant 4 with elevated acylated anthocyanins (Fig. 4D), suggesting a distinct phenolic fingerprint despite its reduced color expression in 2021. Collectively, these findings indicate that vintage conditions and vineyard-specific factors interact to shape wine phenolic composition and color stability, with different compound classes related to color expression under varying environmental scenarios.

### 3.4 Co-variation network analysis of phenolic compounds from berries to wines

To characterize coordinated phenolic variation and potential continuity from grapes to wines, co-variation network analysis was used to examine cross-stage associations among phenolic compounds in each vintage (Fig. 5). Compared with the relatively simple network observed in 2021, the 2020 vintage exhibited a more complex association pattern involving anthocyanins, flavan-3-ols and multiple anthocyanin-derived pigments.

**Fig. 5:**
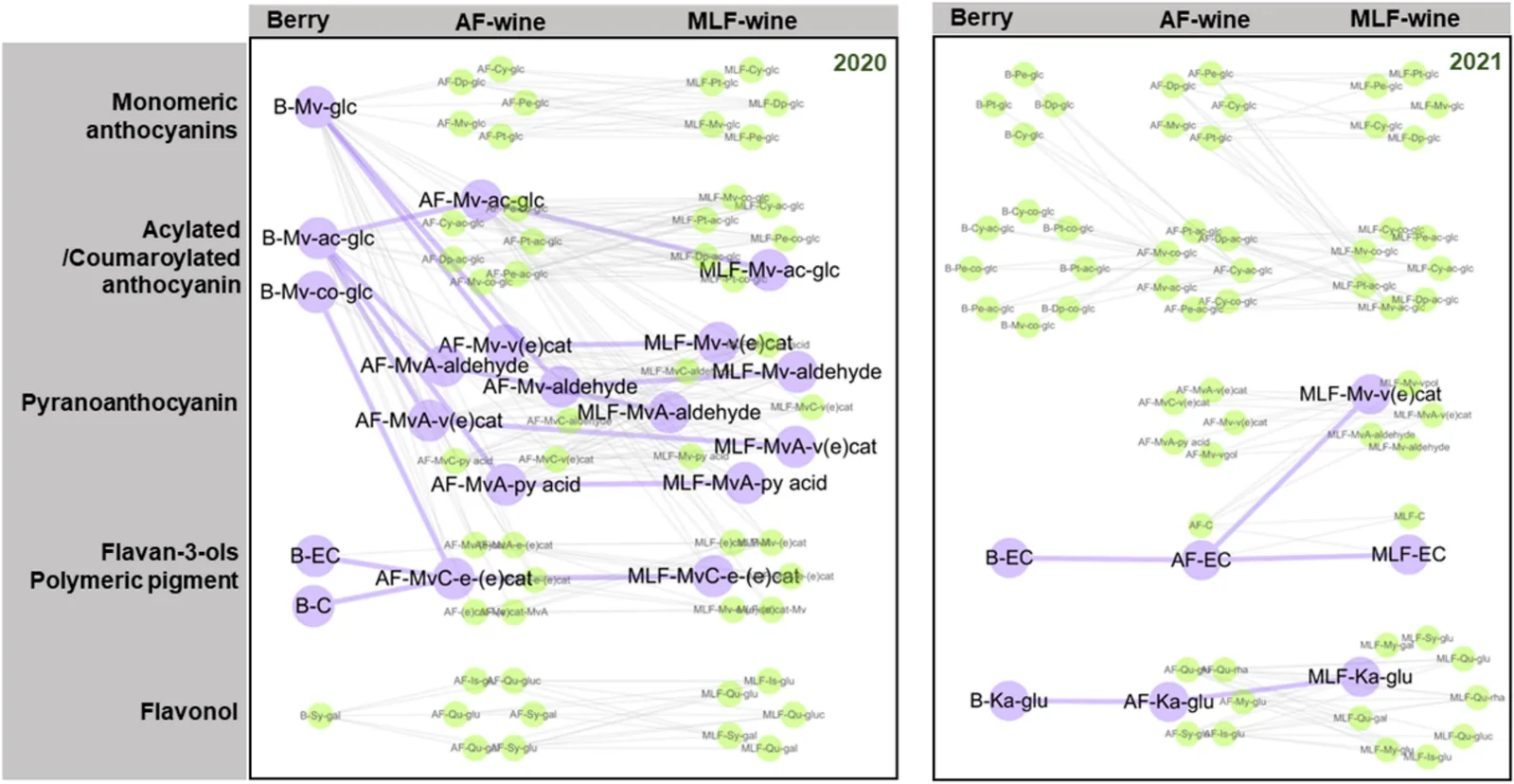
Correlation network illustrating the continuity and transformation of phenolic compounds from grape berries (B) to wines after alcoholic fermentation (AF) and malolactic fermentation (MLF). The network was constructed using pairwise correlation coefficients, and only relationships with *r* > 0.60 were retained. Correlations without a reasonable structural basis or known transformation pathway were excluded. Green nodes represent phenolic compounds connected by at least one retained edge. Purple nodes represent compounds involved in continuous or evolutionarily linked relationships across the berry, AF and MLF stages, highlighting candidate phenolic markers capable of transmitting vineyard plot-specific characteristics throughout the winemaking process. Edges represent retained correlations between compounds across different stages. (For interpretation of the references to color in this figure legend, the reader is referred to the web version of this article.)

In 2020, several network modules were centered on malvidin-based anthocyanins. Although B-Mv-glc (B- represents compounds from grape berries) was not directly associated with its monomeric counterparts in AF or MLF wines, it showed strong positive correlations with MvC-v(*e*)cat and Mv-aldehyde detected at both vinification stages. This pattern suggests that plot-dependent differences in B-Mv-glc were more closely reflected in the formation of anthocyanin-derived pigments than in the persistence of the original monomeric anthocyanin. Similarly, B-Mv-ac-glc was positively associated with AF-Mv-ac-glc and MLF-Mv-ac-glc, indicating continuity throughout vinification. In addition, B-Mv-ac-glc was linked to several derived pigments, including MvA-aldehyde, MvA-py acid and MvA-v(*e*)cat, forming an extensive anthocyanin-centered network. Another prominent module connected B-EC, B—C and B-Mv-co-glc with MvC-e-(e)cat in both AF and MLF wines, highlighting the close association between flavan-3-ols and anthocyanin-derived pigment formation.

By contrast, the 2021 network was characterized by only two major association modules. The first consisted of B-EC, AF-EC and MLF-EC, with AF-EC further associated with MLF-Mv-v(*e*)cat. The second involved Ka-glu, which exhibited strong positive correlations among berry, AF and MLF samples. Compared with 2020, substantially fewer anthocyanin-derived pigments were incorporated into the network, resulting in a simpler association structure dominated by flavan-3-ol and flavonol-related compounds.

Taken together, these results indicate that phenolic associations in 2020 were largely characterized by extensive links between berry anthocyanins and anthocyanin-derived pigments formed during vinification, whereas the 2021 network primarily reflected the continuity of flavan-3-ols and flavonols across stages. The contrasting network structures were consistent with vintage-dependent differences in pigment composition and color expression observed in the resulting wines.

### 3.5 Soil-associated variation in grape phenolic composition

Differences in soil texture, mineral nutrient composition, organic matter content, cation exchange capacity, electrical conductivity, and pH were observed across vineyard plots and soil depths, indicating spatial heterogeneity within the study area (Chen et al., 2023). Based on the PLSR, PLS-DA, and berry-wine correlation network analyses described above, phenolic compounds that were associated with wine color parameters or plot discrimination, or that exhibited continuity during vinification were selected for exploratory soil-association analysis. The soil–phenolic correlations were analyzed separately for each vintage and soil depth using 12 paired observations (four plots × three replicates). Seven phenolic compounds were retained for 2020 and six for 2021 (Fig. 6).

**Fig. 6:**
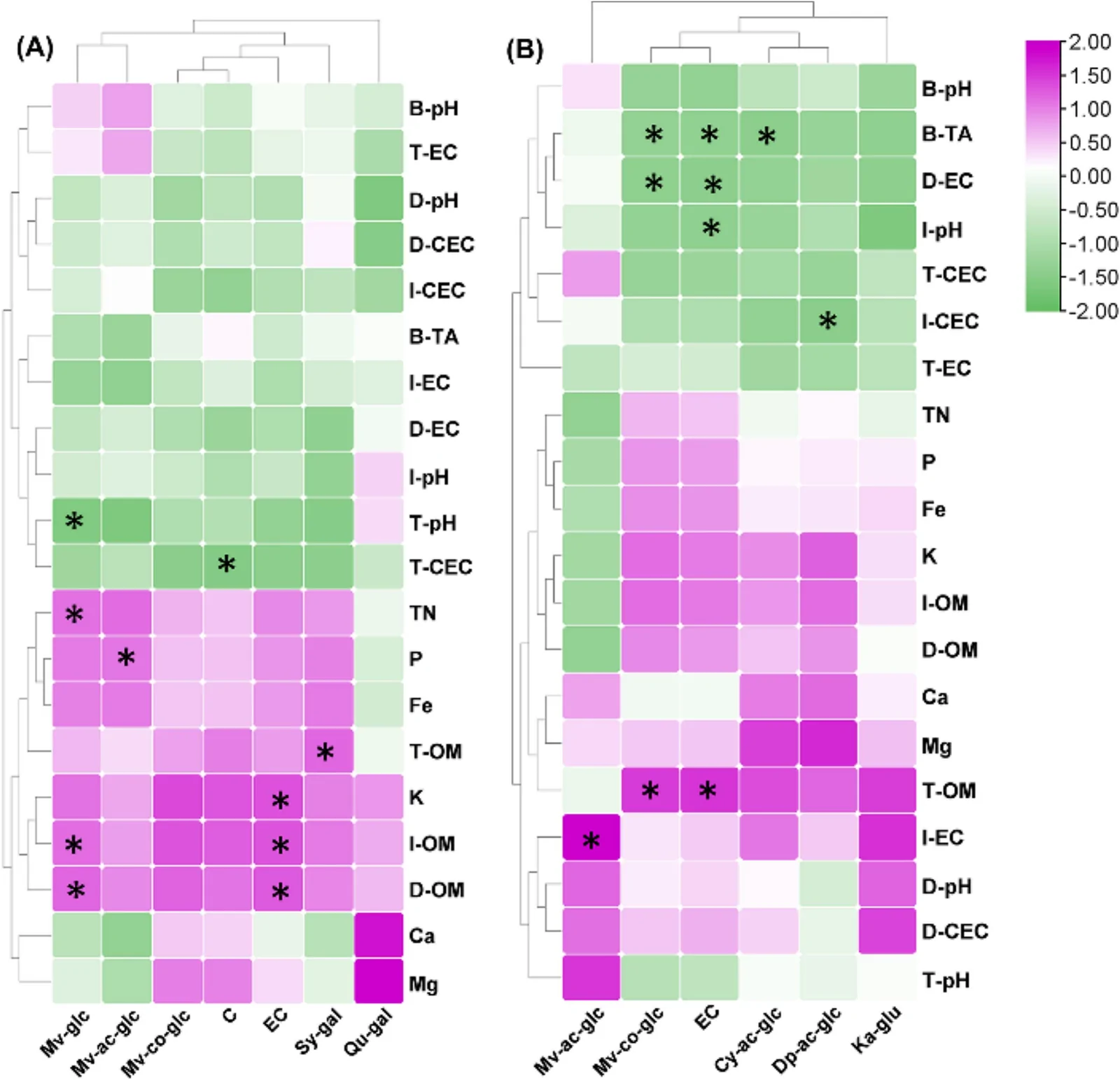
Correlation heatmaps between soil properties and selected grape skin phenolic compounds in the 2020 (A) and 2021 (B) vintages. The phenolic compounds shown were selected based on their contributions to plot discrimination, color expression, and continuity during vinification. Color intensity represents the correlation coefficient between soil variables and phenolic compound concentrations. T-, I-, and D- represent topsoil (0–30 cm), intermediate soil (30–60 cm), and deep soil (60–90 cm), respectively. B- denotes grape berry samples. Soil parameters include electrical conductivity (EC), cation exchange capacity (CEC), organic matter (OM), total nitrogen (TN), and available mineral elements (P, Fe, K, Ca, and Mg). TA indicates titratable acidity. Statistical significance is indicated by *P* < 0.05.

In 2020, clear clustering patterns were observed among soil variables and key berry phenolics. Organic matter content across all soil depths (T-OM, I-OM and D-OM) and total nitrogen (TN) were positively associated with Mv-glc, while the flavan-3-ol EC showed positive correlations with organic matter and potassium availability. In addition, Mv-ac-glc was positively correlated with available phosphorus, whereas Sy-gal was primarily associated with topsoil organic matter. More broadly, organic matter, nitrogen, phosphorus, potassium and iron formed a coherent group that was positively associated with the three major malvidin-derived anthocyanins and their acylated derivatives, two flavan-3-ols (EC and C), and two flavonols (Sy-gal and Qu-gal). In contrast, soil pH, electrical conductivity (EC), cation exchange capacity (CEC), and berry pH tended to show negative relationships with these compounds.

A similar pattern was observed in 2021 despite the substantial differences in climatic conditions between vintages. Both EC and Mv-co-glc were positively correlated with topsoil organic matter, while Mv-ac-glc was positively associated with the electrical conductivity of the intermediate soil layer. Notably, EC, identified as one of the key compounds in the 2021 network analysis, exhibited negative correlations with berry titratable acidity, deep-soil electrical conductivity and intermediate-soil pH. Similar relationships were observed for Mv-co-glc. Among the six key compounds selected in 2021, four anthocyanins and anthocyanin derivatives (Mv-co-glc, Mv-ac-glc, Cy-ac-glc and Dp-ac-glc), together with the flavan-3-ol EC and the flavonol Ka-glu, generally displayed positive associations with organic matter and a tendency toward positive relationships with mineral nutrients including phosphorus, iron, potassium, calcium and magnesium.

Taken together, the results from both vintages revealed broadly consistent associations between soil properties and key phenolic compounds, despite substantial year-to-year differences in climatic conditions, pigment composition, and wine color expression. Organic matter and mineral nutrient availability were generally positively associated with the accumulation of key phenolic compounds, whereas higher soil pH, electrical conductivity, and cation exchange capacity tended to show negative relationships. By simultaneously exhibiting soil responsiveness, plot discrimination capacity, and continuity throughout vinification, these compounds may serve as candidate links between the vineyard environment and the phenolic profiles of berries and wines.

## 4 Discussion

### 4.1 Covariation of phenolic composition and wine color across vineyard plots and vintages

Wine color was not determined by monomeric anthocyanin concentration alone. This was particularly evident in 2021, when the wines generally contained higher concentrations of monomeric anthocyanins than those produced in 2020 but exhibited higher b* and *h*_*ab*_ values and a more yellow-toned appearance. Differences were also evident among plots within the same vintage. For example, despite the comparable total anthocyanin concentrations of CS1 and CS2 in 2021, CS1 exhibited a higher a* value and a lower L* value than CS2. The PLSR analysis suggested compositional associations consistent with these observations: a* and C*_ab_ tended to covary with anthocyanin-derived pigments, whereas b* and *h*_*ab*_ showed closer covariation with flavonols (Fig. 2). Thus, the visual expression of wine color depended on the relative abundance and transformation state of different pigment classes, as well as their interactions with other phenolic compounds, rather than simply on the total concentration of monomeric anthocyanins.

The greater contribution of pyranoanthocyanins and polymeric pigments to persistent wine color can be explained by their structural stability. The additional pyran ring of pyranoanthocyanins protects the chromophore and reduces its susceptibility to hydration, pH-dependent discoloration, and SO₂ bleaching, while the covalent condensation or bridging reactions involved in polymeric pigment formation generate relatively stable-colored structures (He et al., 2012; Zhang et al., 2022). Previous studies have similarly shown that grape-derived monomeric anthocyanins progressively decrease during wine aging, whereas pyranoanthocyanins and other anthocyanin-derived pigments become increasingly important contributors to persistent color (Monagas et al., 2005; Zhang et al., 2021). Our results were consistent with these established patterns. In 2020, CS1 was associated primarily with polymeric pigments, whereas CS3 was closely associated with pyranoanthocyanins; both wines exhibited higher a* and C*_ab_ values (Fig. 2, Fig. 4). In contrast, polymeric pigments were largely below the detection limit in 2021. Nevertheless, CS3 remained closely associated with pyranoanthocyanins and exhibited the highest C*_ab_ value, further supporting the contribution of these stable pigments to color intensity under contrasting vintage conditions.

Total phenolic content is an aggregate measure and does not necessarily represent the abundance of chromatically active pigments. Wines with comparable total phenolic concentrations may contain different proportions of monomeric anthocyanins, pyranoanthocyanins, polymeric pigments, flavonols, flavan-3-ols, and phenolic acids, which differ markedly in their direct contributions to color and their reactivity during vinification. The balance between stable anthocyanin-derived pigments and other phenolic classes may therefore explain the contrasting color patterns among plots and vintages. In 2021, CS1 and CS2 were positioned closer to the b* and *h*_*ab*_ vectors and showed limited accumulation of anthocyanin-derived pigments, whereas flavonols were more closely associated with yellow-toned color attributes. Although flavan-3-ols and phenolic acids contribute relatively little direct red color, they may participate in the formation of derived pigments, while flavonols can further modify wine hue through their own chromatic properties and copigmentation with anthocyanins (Del-Castillo-Alonso et al., 2016; Zhang et al., 2024, 2022). Consequently, differences in pigment composition, polymerization state, and copigmentation balance can produce distinct color characteristics even when total phenolic concentrations are similar. The wetter conditions in 2021 coincided with differences in berry phenolic accumulation and subsequent pigment evolution during vinification. Collectively, vintage- and plot-related variation in phenolic composition was associated with differences in wine color, but these statistical relationships do not establish direct causation or formal mediation by phenolic composition.

### 4.2 Plot discriminating phenolic signatures and their continuity during vinification

The phenolic compounds responsible for vineyard plot discrimination were not uniformly preserved throughout vinification. While PLS-DA identified distinct sets of discriminating compounds in berries, AF wines, and MLF wines, the correlation network analysis demonstrated that a subset of these compounds-maintained continuity across stages, either through direct persistence or through predictable transformation pathways (Fig. 5).

Univariate comparisons, multivariate statistics, and co-variation network analysis provided complementary levels of information. Univariate analysis identified plot-dependent differences in individual phenolic compounds, but considered each compound separately. PLS-DA incorporated the covariance among multiple phenolic variables to evaluate overall plot discrimination and identify compounds with high contributions to that discrimination, while PLSR related the combined phenolic profile to multiple wine color attributes. Co-variation network analysis further introduced a stage-resolved relational dimension by mapping associations among phenolic compounds in berries, AF wines, and MLF wines. This approach distinguished compounds that persisted directly across stages from berry compounds whose plot-dependent variation was associated with the formation of derived pigments, while also highlighting recurrent or highly connected candidate markers. Thus, the combined analyses provided an integrated view of individual differences, overall vineyard discrimination, color-related phenolic patterns, and phenolic continuity during vinification. Nevertheless, the network edges represent statistical co-variation and do not by themselves demonstrate direct biochemical conversion or causality.

Among the different phenolic classes, flavan-3-ols exhibited the greatest stability during vinification. EC consistently showed strong associations between berries, AF wines, and MLF wines in both vintages, indicating that plot-dependent differences established in the vineyard could be retained throughout the winemaking process. The persistence of EC across all matrices suggests that certain flavan-3-ols may serve as robust carriers of vineyard identity and represent stable indicators of terroir expression.

In contrast, anthocyanins displayed a more dynamic behavior. Previous studies have reported substantial losses of monomeric anthocyanins during alcoholic fermentation due to adsorption by yeast cell walls, oxidation, and participation in condensation reactions (Uzkuç et al., 2022; Zhang et al., 2021). Consistent with these observations, several monomeric anthocyanins identified as plot-discriminating compounds in berries were no longer among the most important discriminating variables in the corresponding wines. However, the network analysis revealed that their vineyard-specific signatures were not entirely lost. Instead, these compounds remained connected to anthocyanin-derived pigments detected after fermentation.

The 2020 vintage provided a particularly clear example of this phenomenon. Berry Mv-glc exhibited strong associations with MvC-v(*e*)cat and Mv-aldehyde in both AF and MLF wines, while Mv-ac-glc was linked to multiple anthocyanin-derived pigments, including MvA-aldehyde, MvA-py acid, and MvA-v(*e*)cat. These relationships suggest that vineyard-specific differences initially expressed through monomeric anthocyanins were subsequently incorporated into more stable pyranoanthocyanins and polymeric pigments during vinification. Therefore, the continuity of vineyard signatures was achieved not only through compound persistence but also through pigment evolution.

The nature of these phenolic signatures varied between vintages. In 2020, vineyard discrimination was strongly associated with anthocyanin-derived pigments and their transformation products, reflecting the extensive formation of pyranoanthocyanins and polymeric pigments. In contrast, the 2021 vintage showed a simpler network structure characterized by fewer derived pigments and greater contributions from flavan-3-ols and flavonols. Against this vintage-dependent background, EC, Mv-ac-glc, and Mv-co-glc repeatedly emerged as important markers across analyses, suggesting that they represent complementary mechanisms by which vineyard-specific information may persist during vinification. EC exhibited direct continuity from berries through AF and MLF wines, indicating that plot-dependent differences in this flavan-3-ol were retained across processing stages. By contrast, Mv-ac-glc and Mv-co-glc represented more dynamic signatures: although monomeric anthocyanins were strongly affected by vintage and vinification, these acylated anthocyanins remained associated with plot discrimination, soil properties, and anthocyanin-derived pigments formed during fermentation (Fig. 5, Fig. 6). Their recurrence across multivariate discrimination, berry-to-wine network, and soil-association analyses indicates that vineyard identity may be retained through both direct compound persistence and transformation-linked associations. Compared with an assessment based only on monomeric anthocyanin concentrations, this multi-class and process-based set of markers is less dependent on a single phenolic group or sampling stage and provides a more integrated representation of micro-terroir expression. Nevertheless, because the present study covered only four plots and two vintages, EC, Mv-ac-glc, and Mv-co-glc should be regarded as candidate, relatively stable indicators of vineyard plot identity that require validation across additional vineyards and vintages.

### 4.3 Potential associations between soil factors and grape phenolic composition

Among the soil variables examined in this study, organic matter emerged as the factor most consistently associated with the accumulation of key phenolic compounds. Recent metabolomic analyses of Cabernet Sauvignon have likewise shown that terroir-scale environmental variation is associated with distinct grape polyphenol profiles (Lu et al., 2025). Across both vintages, higher organic matter content was generally associated with increased concentrations of anthocyanins, flavan-3-ols, and flavonols. Soil organic matter can improve soil structure and plant-available water while buffering soil nutrient availability, thereby stabilizing vine water and nutrient status and influencing photosynthetic carbon supply and berry development (Lal, 2020). Microbial activity and organic matter turnover may additionally influence the availability and retention of N, P, K, and micronutrients. Therefore, the observed associations with organic matter probably represent an indirect soil-vine-berry pathway rather than a direct contribution of organic matter to phenolic biosynthesis.

At the biochemical level, anthocyanins, flavonols, and flavan-3-ols originate from the interconnected phenylpropanoid and flavonoid pathways, which depend on phenylalanine and photosynthetically derived carbon skeletons (Vogt, 2010). Soil nutrients may therefore influence phenolic accumulation by modifying precursor availability, carbon–nitrogen allocation, canopy development, and the expression of pathway-related genes. Nitrogen supports amino acid and protein synthesis, but excessive N supply may redirect carbon toward primary growth and increase canopy shading, thereby reducing cluster exposure and potentially suppressing phenolic accumulation. Indeed, cluster-zone leaf removal increased anthocyanin and flavonol concentrations in Cabernet Sauvignon and Marselan berries, supporting the importance of canopy-mediated cluster microclimate in phenolic accumulation (Yao et al., 2024). Moreover, low N supply increased anthocyanin accumulation and the expression of PAL, CHS, F3′5′H, DFR, and LDOX in Cabernet Sauvignon berries (Soubeyrand et al., 2014). Phosphorus participates in cellular energy transfer and metabolic signaling, and phosphate availability has also been shown to alter anthocyanin accumulation and the expression of associated biosynthetic genes in grape cells (Yin et al., 2012). Thus, the positive associations observed here should not be interpreted as simple linear responses of phenolic accumulation to increasing N or P availability.

Potassium may affect berry phenolic composition through its roles in enzyme activation, osmotic regulation, phloem transport, and the translocation of sugars and other assimilates into ripening berries (Mpelasoka et al., 2003). Vineyard fertilization experiments have also demonstrated that N and K supply can alter phenolic composition during grape ripening (Delgado et al., 2004). Iron provides a more direct biochemical connection because flavanone 3-hydroxylase, flavonol synthase, and anthocyanidin synthase/leucoanthocyanidin dioxygenase are Fe (II)- and 2-oxoglutarate-dependent enzymes in the flavonoid pathway (Turnbull et al., 2004). In Cabernet Sauvignon, moderate Fe supply increased anthocyanin accumulation and the expression of CHI, F3H, LDOX, and AOMT, whereas both Fe deficiency and excess reduced anthocyanin concentrations (Shi et al., 2017).

Whereas organic matter and several mineral nutrients were generally positively associated with phenolic compounds, soil pH and electrical conductivity showed predominantly negative relationships. Elevated soil pH may reduce Fe availability, thereby indirectly constraining Fe-dependent steps of flavonoid biosynthesis; several key enzymes in this pathway require Fe (II), and moderate Fe supply has been shown to promote anthocyanin accumulation in Cabernet Sauvignon berries (Shi et al., 2017; Turnbull et al., 2004). Likewise, increased electrical conductivity may reflect soil conditions that alter nutrient uptake and vine physiological responses, ultimately influencing flavonoid biosynthesis. The consistency of these negative associations across vintages suggests that soil chemical conditions may impose relatively persistent constraints on phenolic accumulation. Collectively, these findings suggest that the correlations observed in the present study may reflect combined effects of soil water and nutrient retention, carbon allocation, canopy microclimate, assimilate transport, and enzymatic or transcriptional regulation. Nevertheless, because the soil variables covaried and were not experimentally manipulated, these mechanisms should be regarded as biologically plausible hypotheses rather than evidence of direct causality.

Notably, despite the substantial climatic differences between 2020 and 2021, several soil–phenolic associations recurred across both vintages. In particular, Mv-ac-glc, Mv-co-glc, and the flavan-3-ol epicatechin (EC) were repeatedly associated with soil organic matter and nutrient-related variables. Their recurrence under contrasting vintage conditions suggests that these compounds may show relatively consistent associations with soil organic matter and nutrient status. They may therefore serve as candidate indicators of plot-related relationships between vineyard soil conditions and berry phenolic profiles. Nevertheless, given the relatively limited number of observations, these relationships should be regarded as exploratory and require validation across additional vineyard sites and vintages.

### 4.4 Synthesis, limitations, and future perspectives

Across the two vintages, both vintage and vineyard plot were associated with variation in grape and wine phenolic profiles and wine color. The wetter 2021 vintage coincided with yellower color attributes and markedly lower polymeric pigment abundance, yet plot-associated differentiation remained detectable. Across analyses, wine color was more closely related to pigment composition and transformation state, particularly the occurrence of pyranoanthocyanins and polymeric pigments, than to total monomeric anthocyanin concentration alone. EC, Mv-ac-glc, and Mv-co-glc recurred in analyses of plot discrimination and phenolic continuity from berries to wines. They are therefore proposed as candidate indicators of plot identity but require further validation. Their associations with soil organic matter and nutrient availability remain exploratory and do not establish causal relationships between soil conditions and vine responses.

These interpretations are constrained by the study of four neighboring plots over two vintages, the observational and covarying nature of the soil variables, and the semi-quantitative determination of analytes lacking authentic standards. For some monomeric phenolics and anthocyanin-derived pigments, structurally related reference standards were used, including malvidin-3*-O-*glucoside for anthocyanin-derived pigments. Differences in ionization efficiency and detector response between the target compounds and representative standards may affect the estimated absolute concentrations. Accordingly, relative variation of the same compound across samples is more reliable than absolute comparisons among structurally distinct compounds or phenolic classes. In addition, although all plots were harvested on the same date within each vintage and total soluble solids were comparable at harvest, plot-associated differences in berry weight, pH, and titratable acidity may have contributed to part of the observed variation in phenolic composition. Future work should validate the candidate markers across additional vineyards and vintages, quantify key pigments using authentic standards, and combine longitudinal berry-to-wine sampling with controlled soil, nutrient, or water treatments to test the proposed associations and transformation pathways.

## CRediT authorship contribution statement

**Xiao Meng:** Writing – original draft, Visualization, Validation, Software, Formal analysis, Data curation. **Hualin Zhang:** Methodology, Formal analysis, Data curation. **Xuechen Yao:** Visualization, Methodology, Data curation. **Yibin Lan:** Supervision, Resources, Methodology, Funding acquisition, Conceptualization. **Tonghua Shi:** Validation, Supervision, Resources, Funding acquisition. **Yanzhi Cui:** Validation, Supervision, Project administration, Funding acquisition. **Changqing Duan:** Writing – review & editing, Supervision, Project administration, Conceptualization. **Guangfeng Wu:** Writing – review & editing, Supervision, Project administration, Data curation, Conceptualization. **Qiuhong Pan:** Writing – review & editing, Supervision, Resources, Funding acquisition, Conceptualization.

## Fundings

This work was funded by the 10.13039/501100012166National Key Research and Development Program of China (2023YFF1104203), the Science & Technology Cooperation Project between China Agricultural University and Longus Vineyard Co., Ltd., and the Earmarked Fund for 10.13039/501100010203China Agriculture Research System (CARS-29).

## Declaration of competing interest

The authors declare that they have no known competing financial interests or personal relationships that could have appeared to influence the work reported in this paper.

## Data Availability

Data will be made available on request.
