# Supplementary material for: Micro-scale vineyard variation influences phenolic composition and color expression in ‘cabernet sauvignon’ wines
Source: Food Chem X. 2026 Nov 4;40:104751. doi: 10.1016/j.fochx.2026.104751 (PMC13760702; doi:10.1016/j.fochx.2026.104751)
Supplement: Supplementary file 1 [file mmc1.docx]

**Figure S1. Physicochemical parameters of berry and wine samples across vineyard plots.**

(A) Berry parameters including hundred-berry weight, total soluble solids (°Brix), pH, and titratable acidity; (B) wine parameters after alcoholic fermentation (AF) including ethanol content, residual sugar, pH, and titratable acidity; (C) wine parameters after malolactic fermentation (MLF) including ethanol content, residual sugar, pH, and titratable acidity. Data are presented as mean ± SD (n = 3). One‑way ANOVA with Tukey’s HSD post‑hoc test was applied; different lowercase letters indicate significant differences among plots for each parameter (*p* < 0.05). Purple dots and green squares represent samples from vintage 2020 and vintage 2021, respectively.

**Figure S2. Permutation validation of the partial least squares regression (PLSR) models relating wine phenolic composition to CIELAB color parameters in 2020 (A) and 2021 (B).** Each model was evaluated using 500 permutations. The R² and Q² regression-line intercepts were 0.1587 and −0.6222 for the 2020 model and 0.2256 and −0.3240 for the 2021 model, respectively.
